# *De novo* Migraine with Aura in the Third Trimester of Pregnancy: A Case Report and Literature review

**DOI:** 10.15388/Amed.2021.28.1.19

**Published:** 2021-05-11

**Authors:** Elena Paškevičiūtė, Diana Bužinskienė, Kristina Ryliškienė

**Affiliations:** ORCID: https://orcid.org/0000-0002-1847-5640 Vilnius University, Faculty of Medicine, Vilnius, Lithuania; ORCID: https://orcid.org/0000-0002-4522-0600 Clinic of Obstetrics and Gynaecology, Institute of Clinical Medicine, Faculty of Medicine, Vilnius University, Vilnius, Lithuania Centre of Obstetrics and Gynaecology, Vilnius University Hospital Santaros Klinikos, Vilnius, Lithuania; ORCID: https://orcid.org/0000-0001-9596-1733 Department of Neurology and Neurosurgery, Institute of Clinical Medicine, Faculty of Medicine, Vilnius University, Vilnius, Lithuania

**Keywords:** Migraine with aura, Pregnancy, Secondary headache disorder, Acute headache, Migraine management

## Abstract

**Background:**

Among all headache disorders, migraine has the highest prevalence during gestation. The majority of migraineurs experience improvement during pregnancy, but a few may experience migraine for the first time. This poses a diagnostic challenge in the differential diagnosis between primary and life-threatening secondary headache disorders. Because pregnancy itself is an independent risk factor for secondary headache disorders, it is mandatory to exclude these conditions in order to diagnose migraine. There is a large body of literature about pre-existing migraine course during pregnancy and its link with adverse pregnancy outcomes, but there are no studies examining these aspects among women with new-onset migraine during pregnancy.

**Case report.:**

A 31-year-old female at 33 weeks of gestation (gravida 2, para 2) was referred to the neurologist eds disturbances, which were followed by pressing severe headache, rated as 8 out of 10 on a numeric rating scale and accompanied by dizziness. The headache lasted for one day, and dizziness continued to the following day. The patient was investigated for a secondary headache disorder, but laboratory and neuroimaging results were unremarkable. A migraine with aura was diagnosed. The patient was advised to keep a consistent sleep schedule, maintain regular low physical activity, eat regularly and take magnesium supplementation. The patient was informed about a safe treatment approach in case of an acute attack. At 40 weeks of gestation the patient delivered female newborn, weighing 3750g, with Apgar scores of 8 and 9 (due to a nuchal cord). The postpartum period was uneventful. During the subsequent 4 years, the patient did not experience any recurrent migraine attacks and had no pregnancies.

**Conclusion.:**

In order to diagnose a migraine during pregnancy, exclusion of secondary headache disorders is mandatory. Pregnant migraineur should be regularly monitored for adverse birth outcomes. It is essential to educate patients, provide information about the safe treatment of migraine attacks, and explain nonpharmacological prevention and supplementation benefits.

## Abstract (lt)

***De novo* migrenos su aura pasireiškimas trečiajame nėštumo trimestre: klinikinis atvejis ir literatūros apžvalga:**

**Santrauka. Apžvalga.**

Migrena yra dažniausia galvos skausmo priežastis nėštumo metu. Nors daugumai nėščiųjų migrena gerėja, bet daliai jų migrena nėštumo metu gali pasireikšti pirmą kartą. Tai kelia sunkumų diferencijuojant pirminį galvos skausmą nuo pavojingo antrinio galvos skausmo. Kadangi pats nėštumas yra nepriklausomas antrinio galvos skausmo rizikos veiksnys, naujai atsiradusi migrena diagnozuojama tik paneigus kitas galvos skausmo priežastis. Literatūroje aprašomi būdingi migrenos eigos pokyčiai ir padidėjusi komplikacijų rizika nėštumo metu moterims, kurioms migrena diagnozuota dar iki nėštumo. Deja, nėra tyrimų, kuriuose būtų nagrinėti šie aspektai moterims, kurioms migrena pirmąkart pasireiškė tik nėštumo metu.

**Atvejo aprašymas.**

31 metų pacientė (antras nėštumas, 33 nėštumo savaitės) ambulatoriškai konsultuota neurologo dėl prieš 7-ias ir prieš 4-ias dienas pirmąkart gyvenime įvykusių priepuolių, kurių metu sutrikdavo rega, jutimas ir kalba, vėliau atsirasdavo spaudžiantis stiprus, įvertintas 8 iš 10 pagal skaitmeninę analogijos skalę, galvos skausmas, jo sukeltas svaigulys. Galvos skausmas truko vieną parą, svaigulys apie dvi paras. Dėl įtariamo antrinio galvos skausmo atlikti laboratoriniai tyrimai, galvos magnetinio rezonanso tomografija ir angiografija, pakitimų nenustatyta. Diagnozuota migrena su aura. Pacientei rekomenduota laikytis reguliaraus miego ir mitybos režimo bei palaikyti minimalų fizinį krūvį, vartoti magnio papildus. Pacientė informuota apie saugų ūminio priepuolio gydymo būdą. 40-tą nėštumo savaitę natūraliais gimdymo takais gimė moteriškos lyties, 3 750 g svorio naujagimis, įvertintas 8/9 Apgar balais (dėl aplink kaklą apsivyniojusios virkštelės). Laikotarpiu po gimdymo komplikacijų nebuvo. Nėštumo, gimdymo ir pogimdyminio periodo eiga buvo sklandi. Per kitus ketverius metus pacientei migrenos priepuoliai nesikartojo, taip pat pacientė per šį laiką neturėjo nėštumų.

**Išvados.**

Nėštumo metu naujai atsiradusi migrena nustatoma tik paneigus antrinio galvos skausmo priežastis. Migrena serganti nėščioji turi būti dažniau ir reguliariau stebima dėl padidėjusios nėštumo komplikacijų rizikos. Svarbu pacientes mokyti, suteikti informacijos apie saugų migrenos priepuolio gydymą ir nemedikamentinę profilaktiką.

**Raktažodžiai: **Migrena su aura, nėštumas, pirminis galvos skausmas, ūminis galvos skausmas, migrenos gydymas

## Introduction

According to the latest study of the Global Burden of Disease, a migraine is the second highest cause of years lived with disability for both genders and the first among young women [1]. Among all headache disorders, a migraine, followed by a tension-type headache (TTH), has the highest prevalence during gestation [2], and it is estimated to be around 20% [3].

The majority of migraineurs experience improvement during pregnancy [4]. However, for a few women, migraine, especially migraine with aura (MA), may occur for the first time during gestation [5]. It is thought that the mechanism leading to this phenomenon could be elevated oestrogen levels and/or the lack of oestrogen cycling, which are believed to reduce the threshold for cortical spreading depression [6]. Clinical features of a new-onset migraine may resemble secondary headaches caused by life-threatening conditions and especially those attributed to vascular disorders [7]. More so, pregnancy and migraine, especially MA, are risk factors for preeclampsia, cerebral venous thrombosis, transient ischemic attack (TIA) and stroke [8,9]. For this reason, health care workers often face difficulties diagnosing migraine, as well as predicting its course. While there is little data on a new-onset migraine during pregnancy, we report a description of a patient with *de novo *migraine with aura in late pregnancy and provide a literature review. 

## Case report

A 31-year-old female at 33 weeks of gestation (gravida 2, para 2) was referred to the neurologist with a history of two headache episodes, which she experienced for the first time in her life. Episodes occurred spontaneously, without any apparent trigger, seven and four days ago, respectively. During the episodes the patient experienced visual, sensory and speech disturbances. The visual symptom was defined as gradually increasing bilateral flickering in the right visual field, which lasted thirty minutes. In the next fifteen minutes, the patient experienced difficulties of word finding, followed by fifteen minutes of gradually spreading numbness in the right hand and right cheek. Ten minutes after the numbness ended, the patient developed a right-sided headache, which later spread to both sides of the head. Headache was pressing in quality, rated as 8 out of 10 on a numeric rating scale and accompanied by dizziness and balance loss. Neither vomiting nor photo/phonophobia was present. Ibuprofen 300 mg and acetaminophen 500 mg were partially effective: the headache lasted for one day, and dizziness continued for two days. After the second attack, the patient complained of a remaining discomfort on the right side of the head. Her past medical history was unsignificant.

Physical examination showed tachycardia (102 bpm) and normotension (111/76 mmHg). Neurological examination and fundoscopy was unremarkable with the exception of mild and inconsistent right hypoesthesia of face and right hand. No pathologies were identified during gynaecological examination. Complete blood count, venous glucose and urinalysis were normal, elevated D-dimer level (600 mcg/L, normal values < 250 mcg/L) was interpreted as physiological change during pregnancy. Because of the new-onset headache, subjective sensory deficit and elevated D-dimer levels, it was decided to perform 1, 5 T brain magnetic resonance imaging with angiography (MRI/MRA) without contrast enhancement in order to exclude the acute cerebrovascular pathology. No acute ischemia, haemorrhage, and no changes in intracranial arteries, cerebral veins or sinuses were found. Mild cerebellar tonsillar ectopia of 4 mm and left maxillary sinus retention cyst were incidental findings. 

Migraine with typical aura according to the International Classification of Headache Disorders, 3rd edition (ICHD III) criteria was diagnosed (table 1).

The patient was advised to keep a consistent sleep schedule, maintain low physical activity, eat regularly, and take 600 mg of magnesium citrate a day (for 1–2 months) for migraine prophylaxis. Treatment for acute migraine attack was proposed: to take 1 g of paracetamol as soon as flickering starts. If necessary, the dose can be repeated when the headache continues.

There were no complications during the rest of the pregnancy and at 40 weeks of gestation the patient had spontaneous vaginal delivery. Female newborn, weighing 3750g, with Apgar scores of 8 and 9 (due to a nuchal cord) was delivered. The patient and newborn were discharged 3 days later. The postpartum period was uneventful. 

Migraine with aura subsided with no further recurrence over a 4-year follow-up period. During this period, the patient had no pregnancies.

## Discussion

Acute onset headache, especially when associated with other neurological symptoms, causes anxiety to the pregnant female and requires an urgent evaluation by the doctor. One of these occasions may be a new-onset migraine during gestation. New-onset MA (table 1) is more frequent than de novo cases of migraine without aura (MO) [4,10].

A new-onset MO affects from 1 up to 10% and MA from 10.7 up to 14% of pregnant women [10]. Since there are no biomarkers or specific imaging findings, the diagnosis of migraine mainly relies on the history. Due to less experience with neurological disorders, this task becomes even more difficult for the obstetrician/gynaecologist, who consults pregnant women with an acute headache. A migraine mimics many life-threatening headache disorders [11], which leads to the conclusion that in order to prevent possible harm to the mother or offspring, any severe new-onset headache during gestation demands an urgent and accurate exclusion of secondary headache disorders (SHDs) (table 2). Transient focal symptoms associated with aura may resemble vascular disorder, such as TIA. To complicate the matter, TIAs may present with headache [9], and migraine aura can occur without an accompanying headache [11]. The focal deficits are typically sudden and occur all at once in TIA, however in migraine aura, focal symptoms in most cases gradually spread from one modality to another [11]. The presence of positive rather than negative symptoms supports a diagnosis of migraine aura [12]. Nevertheless, MA is by far the most common disorder underlying transient focal symptoms among pregnant women [9]. Overall, detailed history taking and neurologic examination can lead to more accurate predictions of the underlying causes and prevent unnecessary interventions.

**Table 1.** Clinical features and criteria of migraine with aura [11]

ICHD-III diagnostic criteria	Migraine headache features
A. At least two attacks fulfilling criteria B and C B. One or more of the following fully reversible aura symptoms: visual, sensory, speech and/or language, motor, brainstem, retinal C. At least three of the following six characteristics: 1. at least one aura symptom spreads gradually over ≥ 5 minutes 2. two or more aura symptoms occur in succession 3. each individual aura symptom lasts 5–60 minutes 4. at least one aura symptom is unilateral 5. at least one aura symptom is positive 6. the aura is accompanied, or followed within 60 minutes, by headache D. Not better accounted for by another ICHD-3 diagnosis.	Duration: 4–72 hours
	Location: unilateral
	Quality: pulsating
	Pain intensity: moderate to severe
	Aggravating factors: physical activity
	Associated symptoms: nausea and/or vomiting, photophobia and phonophobia

There is no high evidence data on the typical time of the occurrence of *de novo* migraine or on its course after and during the pregnancy. Therefore, it could be a subject for the future studies.

The occurrence of SHDs ranges from 14.3 to 52.6% of all pregnant women presenting with an acute headache [10,18,19]. Altogether, these studies suggest that hypertensive disorders, such as preeclampsia [18] and infections (most commonly viral infections and sinusitis) [4] are the most common causes of SHDs. It is important to emphasize that the hypercoagulable state caused by pregnancy [2,10] and migraine [20] are independent risk factors for preeclampsia, cerebral venous thrombosis, TIA and stroke [8,9]. Two retrospective studies reported that specific clinical findings, so-called “red flags” (Table 3) [4,18], should alarm doctors to investigate for the underlying cause of SHD. 

The majority of cerebrovascular disorders have overlapping presentations but distinctive imaging. This is where the medical team faces another obstacle since pregnancy limits the use of neuroimaging techniques. MRI would be the preferred method. However, head computed tomography is faster and considered safe for the foetus, given its low ionization dose (<1 rad) [21]. Contrast materials should be avoided unless absolutely necessary [22]. Examination of our patient raised suspicion that secondary headache disorder was the underlying cause of the headache. The patient’s history and examination revealed two red-flags: lack of a history of primary headaches and abnormal neurologic examination (sensory deficit). Hence, head MRI/MRA without contrast enhancement was performed to identify possible intracranial pathology. After excluding SHDs, clinical features of the headache can help differentiate between primary headache disorders and are usually enough to diagnose them. In our case headache characteristics resembled TTH rather than a typical migraine. However, full clinical picture should be evaluated. According to the ICHD III, migraine aura is sometimes associated with a headache that does not fulfill criteria provided in Table 1, but this is still regarded as a migraine headache because of its relation to the aura [11]. In other cases, migraine aura may occur without headache.

**Table 2.** Clinical features of secondary headache disorders in pregnancy [11,13–17]

	Headache disorder	Character	Aggravating factors	Other symptoms/signs	Relevant history	Diagnostic method
**Cerebrovascular Disorders**	**Ischaemic stroke**	Ipsilateral or bilateral, variable in quality, moderate	-	FNS, impaired consciousness	Preeclampsia/eclampsia, hyperemesis, multiparity, caesarean delivery, migraine, underlying prothrombotic condition	Non-contrast-enhanced CT scan
	**Cerebral venous thrombosis**	Diffuse, can be unilateral, sudden (even thunderclap) or subacute, progressive, mild to severe	-	FNS, nausea, papilledema, altered mental status, pulsatile tinnitus	Traumatic delivery or caesarean section, postdural puncture CSF leak, migraine, underlying prothrombotic condition, obesity, prolonged bed rest, dehydration, anaemia	MR angiography
	**Reversible cerebral vasoconstriction syndrome **	Diffuse, thunderclap, throbbing, severe, recurring over 1–2 weeks	Straining, exertion	Fluctuating FNS, hypertension	Preeclampsia, postpartum state, hypertensive encephalopathy, use of vasoactive medications/drugs	Conventional cerebral angiography (segments of arterial constriction and dilatation)
**Pituitary apoplexy**	**Pituitary apoplexy**	Retro-orbital, sudden or thunderclap, severe	-	Diplopia, visual field deficits, or decreased visual acuity, vomiting, impaired consciousness, hypopituitarism	Pre-existing pituitary adenoma	MRI
**Space-occupying lesions**	**Intracranial neoplasm**	Diffuse (can be ipsilateral), progressive, mild to severe	Valsalva-like manoeuvres, horizontal position, worsening of neoplasm	FNS, visual changes, nausea/vomiting	Pre-existing pituitary adenoma, meningioma, primary glial brain tumor, colloid cysts or Chiari malformations	MRI
**Pressure-related disorders**	**Idiopathic intracranial hypertension**	Diffuse and/or constant (non-pulsating), daily	Coughing, straining	Visual field defect, pulsatile tinnitus, 6th nerve palsy, papilledema	Obesity, rapid weight gain	Lumbar puncture: opening pressure >250 mmHg
**Hypertensive disorders**	**Preeclamptic **	Diffuse, acute, pulsating	Physical activity, rising blood pressure	Visual changes, epigastric pain, nausea/vomiting, low amount of urine, liver problems, thrombocytopenia, intrauterine growth restriction	>20 weeks of gestation, migraine	Protein/creatinine ratio > 0.3 and SBP ≥140 or DPB ≥ 90 mmHg on two occasions at least 4 hours apart

FNS – focal neurological signs (neurological deficits or seizures), SBP/DPB – systolic/diastolic blood pressure, CT – computer tomography, MRI – magnetic resonance imaging, CSF – cerebrospinal fluid.

**Table 3.** A FALSE PACT: A mnemonic for red flags for secondary headache in pregnancy

	Red flag
**A **	Abnormal neurologic examination
**F **	Fever
**A **	Advancing pain
**L **	Lack of a history of primary headaches
**S **	Seizures
**E **	Elevated blood pressure
**P **	Proteinuria
**A **	Abnormal lumbar puncture or neuroimaging results
**C **	C-reactive protein is higher than normal
**T **	Thrombocytopenia/thrombocytosis or elevated Transaminases

A migraine itself rather than its treatment is also associated with an increased risk of adverse perinatal outcomes. A systematic study confirmed that migraineurs have an increased risk of preterm birth and low birth weight baby by 1.72-fold and 1.8-fold respectively, compared to nonmigraineurs [3]. However, there is a hypothesis that low birth weight may be the consequence of comorbid conditions rather than the migraine itself [23]. Pregnant migraineurs also have an increased risk of preeclampsia, need for the Caesarean section, gestational hypertension [3], placental abruption [24], and even stroke [25]. Unfortunately, there is no data reporting a link between new-onset migraine and adverse pregnancy outcomes.

After diagnosing migraine, doctors face another challenge posed by the limited use of drugs in migraine management during pregnancy. Migraine must be treated adequately since it majorly interferes with daily activities. Treatment may reduce migraine-related presenteeism, absenteeism and improve both physical and mental health during and after delivery. Moreover, vomiting can lead to dehydration and electrolyte imbalances [6]. An abortive treatment for migraine includes acetaminophen, nonsteroidal anti-inflammatory drugs (during second trimester only) as first-line or triptans as second-line choices, and a rule, as with all acute medications during pregnancy, follows: treatment should be used for the shortest period possible to minimize any potential risk [26,27]. Acute and preventative treatment, such as peripheral nerve blocks with lidocaine or ropivacaine is considered safe [28]. However, safety of supraorbital nerve stimulator and transcranial magnetic stimulators has not been formally studied [6]. Antiemetic drugs (metoclopramide as first-line) can be used to relieve symptoms and aid in faster absorption of abortive oral medications [26]. Nonpharmacologic preventative measures are the most important, given the teratogenicity of the majority of migraine therapeutics. Lifestyle and behavioral modifications include regular physical activity, proper hydration, sleep hygiene, avoidance of fasting and certain foods, such as chocolate, red wine, excessive caffeine (abrupt discontinuation should also be avoided) [29]. Regular relaxation training and biofeedback can be helpful [30], and the effect can be enhanced if used in combination with pharmacologic treatment [31]. There is a lack of evidence to determine magnesium supplementation effectiveness in preventing migraines [32]. Preventive pharmacologic treatment, such as propranolol (first-line), amitriptyline and coenzyme Q10 (second-line), are considered safe [6]. 

## Conclusions

A new-onset severe headache during pregnancy requires urgent evaluation to exclude threatening secondary causes, and this is mandatory in order to diagnose a primary headache disorder. A migraine is the most common primary headache among pregnant women. Pregnancy may require closer surveillance for adverse birth outcomes. The symptoms that might signify this need to be explained, and the attendance at regular antenatal checks encouraged. Nonpharmacological preventive measures remain the cornerstone method in preventing the recurrence of migraine. There are too few studies investigating *de novo* migraine during pregnancy, and there are none that analyse different pregnancy outcomes depending on which new-onset migraine subgroup (MA or MO) occurred.
